# The (pro)renin receptor: a novel biomarker and potential therapeutic target for various cancers

**DOI:** 10.1186/s12964-020-0531-3

**Published:** 2020-03-06

**Authors:** Juan Wang, Akira Nishiyama, Makoto Matsuyama, Zhiyu Wang, Ying Yuan

**Affiliations:** 1 Department of Medical Oncology, The Second Affiliated Hospital of Zhejiang University School of Medicine, Hangzhou, China; 2 Cancer Institute (Key Laboratory of Cancer Prevention and Intervention, China National Ministry of Education), The Second Affiliated Hospital, Zhejiang University School of Medicine, 88 Jiefang Road, Hangzhou, 310009 China; 3 Department of Pharmacology, Faculty of Medicine, Kagawa University, Kagawa, Japan; 4 Division of Molecular Genetics, Shigei Medical Research Institute, Okayama, Japan; 5 Department of Immuno-oncology, Fourth Affiliated Hospital of Hebei Medical University, Shijiazhuang, China

**Keywords:** (Pro) renin receptor, Cancer, Wnt/β-catenin signaling pathway, RAS, Biomarker, Cancer targeted therapy

## Abstract

**Background:**

The (pro) renin receptor ((P)RR) plays important roles in various pathways, such as the Wnt/β-catenin, renin-angiotensin system (RAS), MAPK/ERK and PI3K/AKT/mTOR pathways, that are involved in a wide range of physiological and pathological processes incorporating the tumorigenesis. However, our knowledge about (P) RR was mostly limited to its roles in cardiovascular and renal physiological functions and diseases. In the past 5 years, however, compelling evidence has revealed that (P) RR is aberrantly expressed in and contributes to the development of various cancers by different means. For instance, (P) RR was recently demonstrated to induce the oncogenesis of pancreatic, colorectal and brain cancers via the Wnt signaling, while promote the endometrial cancer and glioblastoma through the RAS.

**Methods:**

Combining with the deep analysis of big data from The Cancer Genome Atlas (TCGA) and Genotype-Tissue Expression (GTEx) databases, this review updates and summarizes the recent studies about the newly recognized roles of (P) RR in the pathophysiological processes of cancer development and its detailed functions through related pathways, as well as the novel research progress of (P) RR in related fields including the development and application of soluble (P) RR detection kit and monoclonal (P) RR antibody.

**Results:**

This review provides an overview of the essential roles of (P) RR in the tumorigenesis and progression of various cancers and offers a translational outlook for the future research and clinical practices.

**Conclusion:**

(P) RR in the tumor tissues and/or body fluids of patients may be a novel and promising biomarker and potential therapeutic target for diagnosis, treatment and prognosis prediction in various cancers.

Video Abstract

**Graphical abstract:**

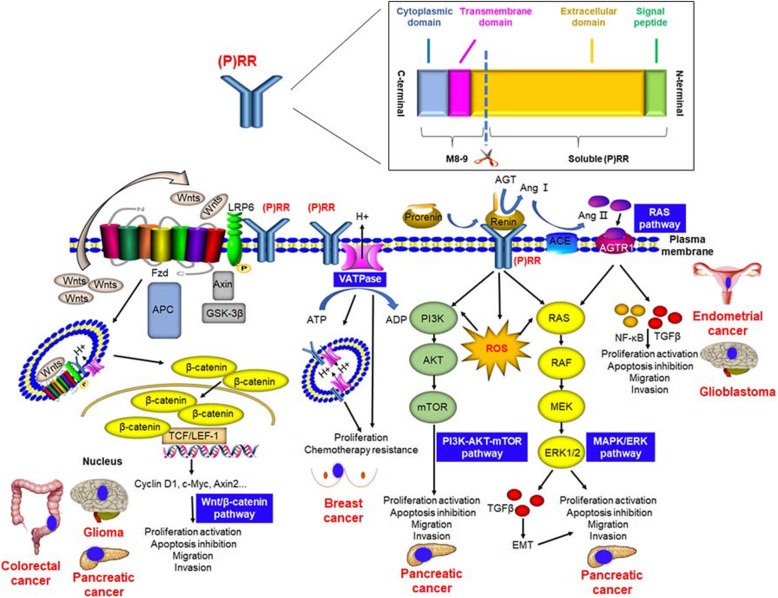

## Background

**Fig. 1:**
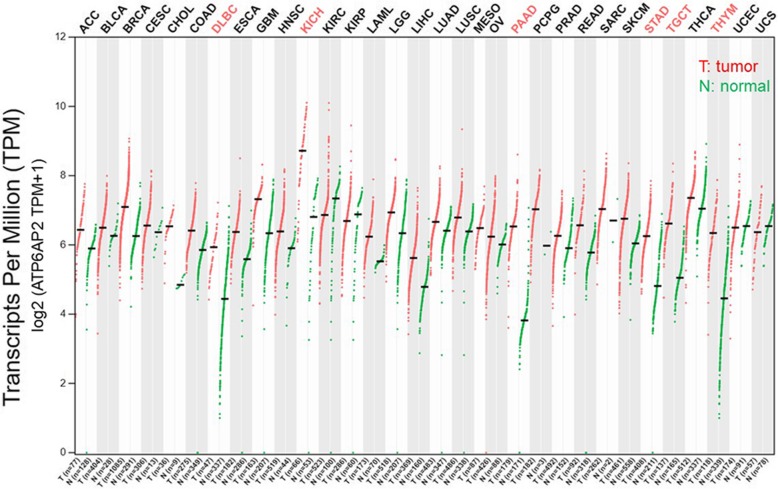
Levels of transcripts of *ATP6AP2* ((P) RR encoding gene) in pan-cancers and corresponding normal tissues. Obviously higher *ATP6AP2* expression was found widely exists in tumor (T) tissues of various cancers compared to the corresponding normal (N) tissues, especially in the lymphoid neoplasm diffuse large B-cell Lymphoma (DLBC), kidney renal clear cell carcinoma (KIRC), pancreatic adenocarcinoma (PAAD), stomach adenocarcinoma (STAD), testicular germ cell tumors (TGCT) and thymoma (THYM). T: tumor tissue; N: normal tissue; *n*: number; ACC: adrenocortical carcinoma; BLCA: bladder urothelial carcinoma; BRCA: breast invasive carcinoma; CESC: cervical squamous cell carcinoma and endocervical adenocarcinoma; CHOL: cholangiocarcinoma; COAD: colon adenocarcinoma; DLBC: lymphoid neoplasm diffuse large B-cell lymphoma; ESCA: esophageal carcinoma; GBM: glioblastoma multiforme; HNSC: head and neck squamous cell carcinoma; KICH: kidney chromophobe; KIRC: kidney renal clear cell carcinoma; KIRP: kidney renal papillary cell carcinoma; LAML: acute myeloid leukemia; LGG: brain lower grade glioma; LIHC: liver hepatocellular carcinoma; LUAD: lung adenocarcinoma; LUSC: lung squamous cell carcinoma; MESO: mesothelioma; OV: ovarian serous cystadenocarcinoma; PAAD: pancreatic adenocarcinoma; PCPG: Pheochromocytoma and Paraganglioma; PRAD: prostate adenocarcinoma; READ: rectum adenocarcinoma; SARC: sarcoma; SKCM: skin cutaneous melanoma; STAD: stomach adenocarcinoma; TGCT: testicular germ cell tumors; THCA: thyroid carcinoma; THYM: thymoma; UCEC: uterine corpus endometrial carcinoma; UCS: uterine carcinosarcoma

The (pro) renin receptor ((P)RR) is a single transmembrane protein consisting of 350 amino acids and encoded by the *ATP6AP2* gene located on the X chromosome. (P) RR is widely expressed in the brain, heart, liver, pancreas, placenta and kidney. Initially, our knowledge about this receptor was limited to its effects on enhancing the tissue renin-angiotensin system (RAS) via binding to its ligands renin and/or prorenin and inducing the activation of intracellular MAPK/ERK (MAPK and ERK are different names of a same protein molecule) pathway (also known as the Ras-Raf-MEK-ERK pathway) independent of the RAS, thus exerting pivotal effects in cardiovascular and renal functions and diseases [1]. (P) RR was later revealed to participate in a wide range of physiological and pathological processes and pathways such as vacuolar H + -ATPase (V-ATPase) function [2] and the Wnt/β-catenin signaling pathway [3]. Interestingly, accumulating studies indicate that the RAS [4], MAPK/ERK [5–7], V-ATPase-related [8] and Wnt/β-catenin signaling [9] pathways contribute to cancer initiation and progression through different means. Considering these connections, scientists asked the following question “Does (P) RR play a role in cancer development through one or several of these mechanisms?” In the past 5 years, compelling evidence has revealed that (P) RR expression is significantly increased in many human cancers and benign tumors, such as colorectal cancer (CRC) [10], pancreatic ductal adenocarcinoma (PDAC) [11, 12], glioma [13], breast carcinoma [14] and aldosterone-producing adenoma [15], in comparison to that in normal tissues. Consistently, we have compared the levels of *ATP6AP2* transcripts in tumor tissues of different cancers and corresponding matched normal tissues, based on the data provided in The Cancer Genome Atlas (TCGA) and Genotype-Tissue Expression (GTEx) databases, and found that obviously higher *ATP6AP2* expression widely exists in various cancers, especially in the lymphoid neoplasm diffuse large B-cell Lymphoma (DLBC), kidney renal clear cell carcinoma (KIRC), pancreatic adenocarcinoma (PAAD), stomach adenocarcinoma (STAD), testicular germ cell tumors (TGCT) and thymoma (THYM) (Fig. 1). This review further summarizes the current knowledge of (P) RR along with the related mechanisms and discusses its translational potential in the context of cancer development, diagnosis, severity evaluation, treatment and prognosis prediction.

### Molecular structure

**Fig. 2:**
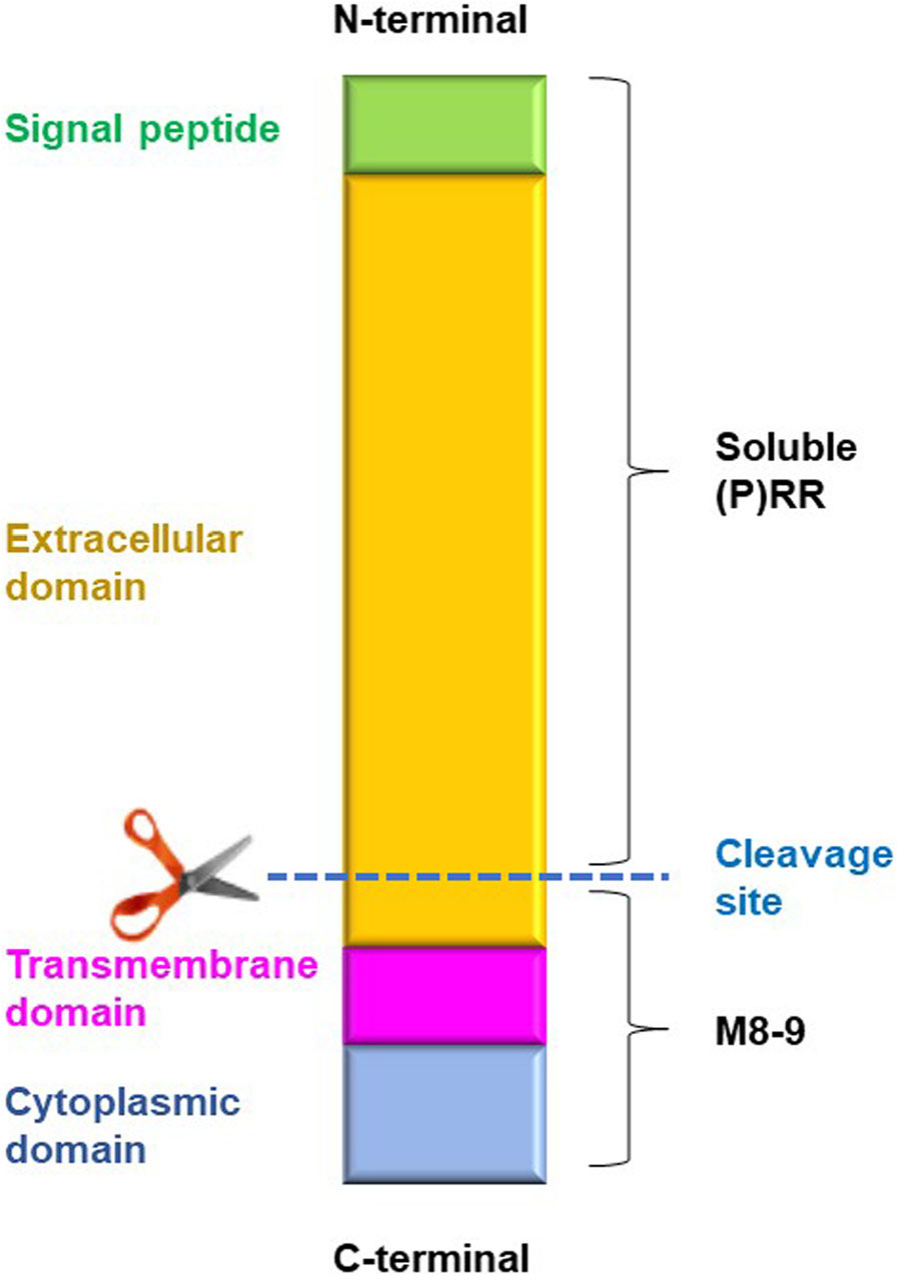
Structure of (P)RR. The (pro) renin receptor ((P)RR) is a single transmembrane protein composed of an extracellular domain containing the N-terminal region, a transmembrane domain and a cytoplasmic domain containing the C-terminal region. The full-length (P) RR (FL(P)RR) protein can be cleaved by furin or ADAM19 into a truncated soluble form containing the N-terminal region (soluble (P)RR; s(P)RR) and a truncated transmembrane form containing the C-terminal region (M8–9 fragment)

(P) RR is composed of an extracellular domain containing the N-terminal region, a transmembrane domain and a cytoplasmic domain containing the C-terminal region. Biologically, the full-length (P) RR (FL(P)RR) protein can be proteolytically cleaved by furin [16] or ADAM19 [17] in the Golgi apparatus to generate a truncated soluble form containing the N-terminal region (soluble (P)RR; s(P)RR) [16] and a truncated transmembrane form containing the C-terminal region (M8–9 fragment) [18] (Fig. 2). s(P) RR is then secreted into body fluids.

### (P) RR and cancer-related pathways or factors

#### The Wnt/β-catenin signaling pathway

**Fig. 3:**
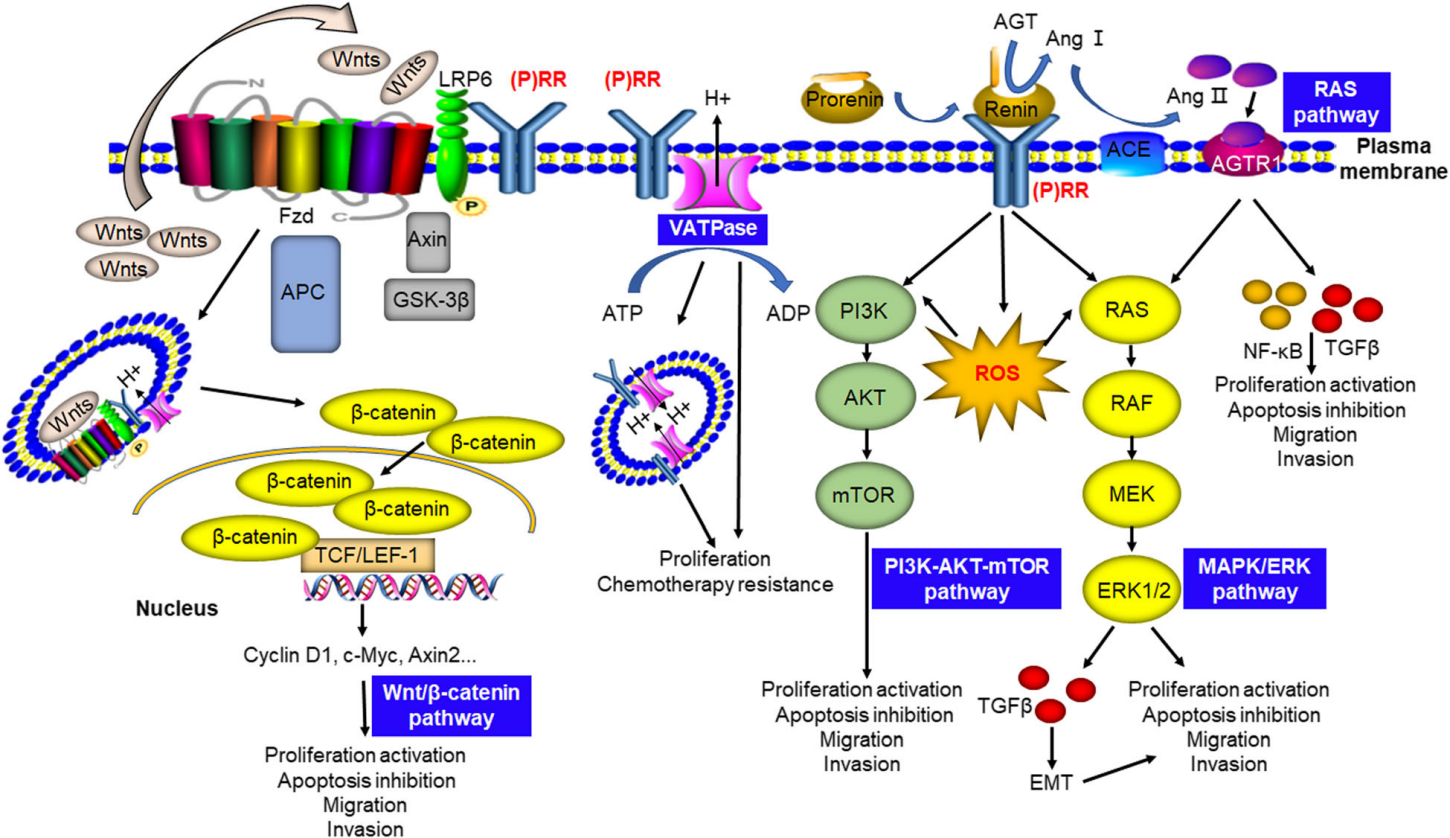
Schematic diagram of the roles of (P) RR in pathways that contribute to oncogenesis, as well as cancer progression and metastasis. In the Wnt/β-catenin pathway, (P) RR is an important component of the Wnt receptor complex and acts as an adaptor between LRP6 and V-ATPase, thus facilitates the binding of Wnt ligands (Wnts) to the Wnt receptor complex, internalization of the complex as a signalosome and Wnt signaling transduction. Full-length (P) RR or M8–9 fragment also interacts with the V-ATPase, on both plasma and vesicle membranes, thus assists it to generate a proton gradient across the plasma membrane that is crucial for LRP6 phosphorylation and subsequent β-catenin activation, as well as to acidize of the vesicles. On binding with the prorenin/renin, (P) RR further contributes to signal transduction of the PI3K/AKT/mTOR and MAPK/ERK pathways, directly or through the induction of reactive oxygen species (ROS) formation. The binding of prorenin or renin to (P) RR enhances their enzymatic activity, which further facilitates the catalysis of angiotensinogen (AGT) to angiotensin (Ang) I. Then, Ang I will be acted on by angiotensin-converting enzyme (ACE) to produce Ang II, which triggers Ang II receptor-mediated signal transduction, leading to elevated tissue RAS activity as well as the formation of NF-κB and transforming growth factor (TGF)β

The Wnt/β-catenin pathway is well known for its essential contributions to the initiation of various cancers such as colorectal [19], gastric [20], prostate [21], breast [22] and adrenocortical [23] cancer. Without the binding of Wnt ligands (Wnts) to the Wnt receptor complex (initially thought to be composed only of Frizzled and LRP5/6), several proteins including APC, Axin and GSK3, usually combine and form the ‘destruction complex’ to cause β-catenin inactivation, ubiquitination and degradation. The binding of Wnts to the Wnt receptor complex leads to phosphorylation of LRP6, internalization of the complex as an LRP6 signalosome and following disruption of the destruction complex, thus protecting β-catenin from inactivation and degradation. Active β-catenin trans-locates to the nucleus and binds the transcription factor TCF/LEF to enhance the expression of target oncogenes such as *c-Myc, AXIN2* and *CCND1* (which encodes Cyclin D1) [24–26]. The first link between (P) RR and the Wnt/β-catenin pathway was clarified by Cruciat et al. [3], who indicated that (P) RR is an important component of the Wnt receptor complex and acts as an adaptor between LRP6 and the V-ATPase independent of the RAS, thus facilitating the binding of Wnts to the Wnt receptor complex [3]. Based on this evidence, further studies convincingly revealed that (P) RR promotes pancreatic [11], brain [13] and colorectal [10] cancers through the Wnt/β-catenin pathway. Interestingly, research also suggests that (P) RR not only serves as a membrane adaptor protein but also exists in the cytoplasm and positively affects the protein expression level of Wnt2 in glioma cells [13] as well as that of Wnt3 and total LRP6 in CRC cells [10]. In summary, (P) RR is a potential novel onco-protein in Wnt/β-catenin pathway-related oncogenesis (Fig. 3).

#### The RAS pathway

The most well-known function of (P) RR is in the RAS. The binding of renin or its precursor prorenin to (P) RR enhances the enzymatic activity of these molecules, which further facilitates the catalysis of angiotensinogen (AGT) to angiotensin (Ang) I. Then, Ang I is acted on by angiotensin-converting enzyme (ACE) to produce Ang II, which triggers Ang II receptor-mediated signal transduction, leading to elevated tissue RAS activity [4]. Accumulating evidence suggests that the RAS affects cancer cell proliferation, apoptosis inhibition, migration, and invasion, as well as metastasis [27]. The increased activation of the RAS through both Ang II/Ang II receptor (AGTR) 1 and (pro)renin/(P) RR promotes cancer development by stimulating the downstream factors that contribute to oncogenesis [28]. Interestingly, transforming growth factor (TGF) β, which is crucial for epithelial-to-mesenchymal transition (EMT) of metastatic cancer cells and is positively associated with RAS activity, is commonly upregulated in tumor tissues [29], which suggesting that (P) RR might promote cancer metastasis through RAS-mediated TGFβ activation. Therefore, (P) RR may promote cancer initiation and progression via the RAS (Fig. 3).

#### The MAPK/ERK and PI3K/AKT/mTOR pathways

The binding of renin or prorenin to (P) RR activates MAPK/ERK signaling and up-regulates extracellular signal-regulated kinase 1/2 (ERK1/2) in several types of cells, such as collecting duct cells, monocytes, mesangial cells, and neurons [30]. ERK1/2 activation enhances cell proliferation and induces TGF-β generation [6, 7], which mediates the pathogenesis and metastasis of cancer. Additionally, (P) RR induces reactive oxygen species (ROS) formation, which is independent of Ang II, thus further contributing to MAPK/ERK and PI3K/AKT/mTOR signal transduction [31]. Consistent with these observations, Arundhathi et al. [32] investigated the function of (P) RR in the biological activities of the human pancreatic cancer cell lines Panc-1 and ASPC and found that (P) RR exerts a cancer-promoting effect by enhancing activity in the MAPK/ERK and PI3K/AKT/mTOR signaling pathways. In particular, the data from this group showed that (P) RR overexpression increased the phosphorylation of AKT, ERK1/2, and mammalian target of rapamycin (mTOR) and elevated the level of NF-κB; however, (P) RR silencing downregulated the expression of ERK1/2, AKT and NF-κB [32] in pancreatic cancer cells. Taken together, these findings indicate that (P) RR may contribute to cancer development through the MAPK/ERK and PI3K/AKT/mTOR pathways (Fig. 3).

#### The V-ATPase

The V-ATPase actively transfers protons into vesicles including lysosomes, endosomes and autophagosomes, by consuming energy acquired by the hydrolysis of ATP to ADP, thus acting as a proton pump that is essential for cellular acidification [8]. The V-ATPase is also functionally expressed on the cell membranes of several types of human tumor cells such as promyelocytic leukaemia HL-60 cells and leiomyosarcoma cells [33, 34] as well as breast cancer cells [35]. In addition, bafilomycin A1, an inhibitor of V-ATPase activity, blocked the autophagy and growth of breast carcinoma cells [35]. Moreover, the expression of V-ATPases was elevated in cisplatin-resistant cell lines derived from various human cancer cells [36]. Notably, a truncated, transmembrane portion of (P) RR has been revealed to be associated with V-ATPase function [37]. In addition, Wnt binding stimulates the endocytosis of the signaling complex (including Wnts, Frizzled, LRP6, (P) RR, and the V-ATPase). The V-ATPase, via interaction with (P) RR, then generates a proton gradient across the vesicle membrane that is crucial for LRP6 phosphorylation and subsequent β-catenin activation [37]. These findings suggest that (P) RR might function in cancer promotion by acting synergistically with the V-ATPase (Fig. 3).

### Roles of (P) RR in various cancers

#### PDAC

**Fig. 4:**
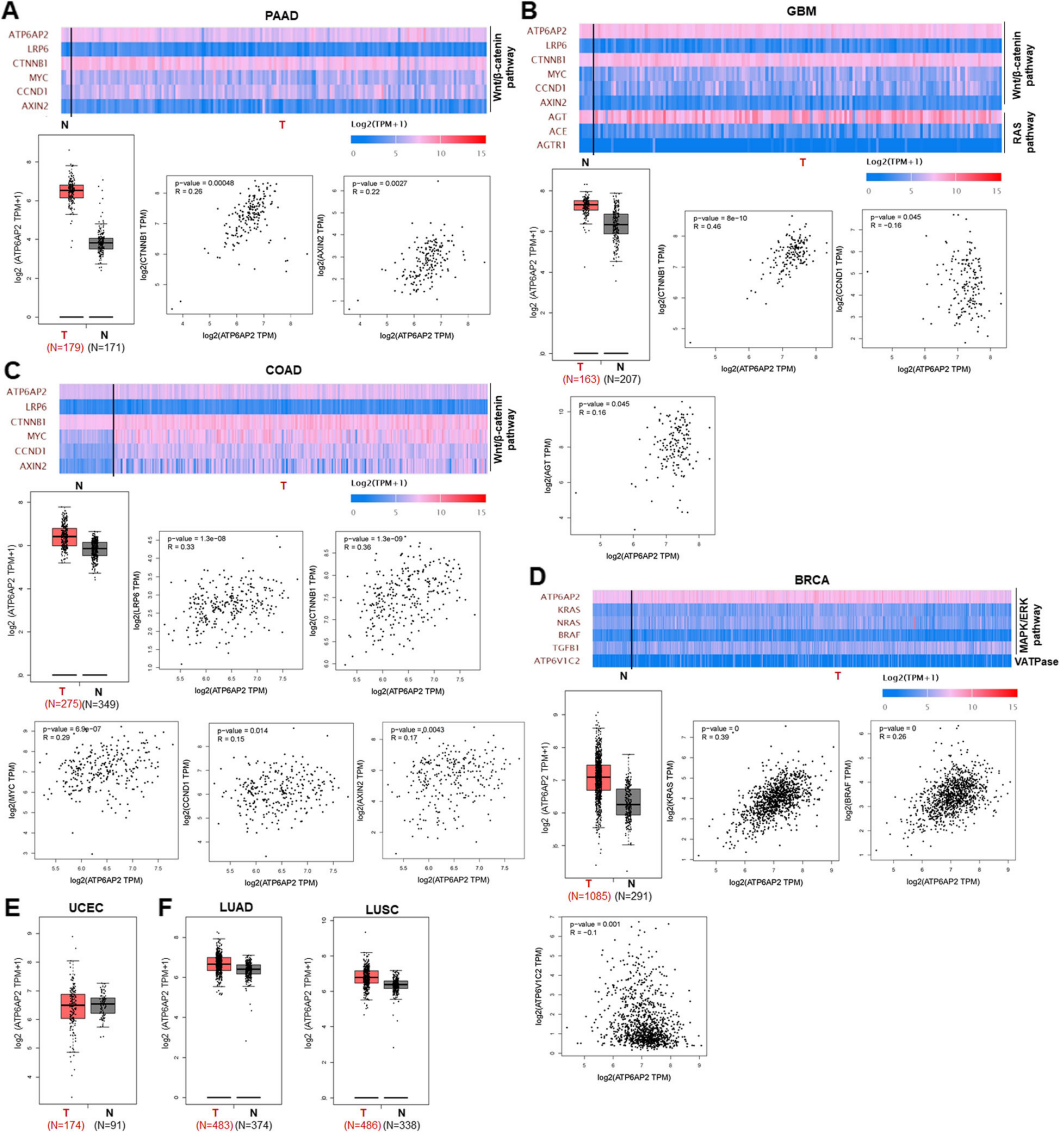
Bioinformatic analysis of the levels of transcripts of *ATP6AP2* ((P) RR encoding gene) in various cancers, the possible cancer-related pathways that (P) RR may be involved in, as well as the correlation between (P) RR and corresponding pathway components, based on data in the TCGA and GTEx databases. **a** Levels of *ATP6AP2* transcripts in tumor tissues of pancreatic adenocarcinoma (PAAD) and normal pancreatic tissues. *ATP6AP2* expression is significantly higher in tumor tissues than that in normal tissues, moreover, level of *ATP6AP2* expression in PAAD is positively correlated with the expression level of *CTNNB1* (β-catenin encoding gene) and Wnt target gene *AXIN2*. **b** Levels of *ATP6AP2* transcripts in tumor tissues of glioblastoma (GBM) and normal matched tissues. *ATP6AP2* expression is higher in tumor tissues than that in normal tissues, moreover, level of *ATP6AP2* expression in GBM is positively correlated with those of *CTNNB1, CCND1* and *AGT.***c** Levels of *ATP6AP2* transcripts in tumor tissues of colon adenocarcinoma (COAD) and normal matched tissues. *ATP6AP2* expression is higher in tumor tissues than that in normal tissues, moreover, level of *ATP6AP2* transcripts in COAD is positively correlated with those of *LRP6*, *CTNNB1, MYC, CCND1* and *AXIN2.***d** Levels of *ATP6AP2* transcripts in tumor tissues of breast invasive carcinoma (BRCA) and normal matched tissues. Level of *ATP6AP2* transcripts in tumor tissues is obviously higher than that in normal tissues, moreover, level of *ATP6AP2* expression in BRCA is positively correlated with those of the MAKP/ERK pathway components *KRAS* and *BRAF*, as well as that of the V-ATPase encoding gene *ATP6V1C2.***e** Expression of *ATP6AP2* in tumor tissues of uterine corpus endometrial carcinoma (UCEC) is at the similar level to that of the matched normal tissues. **f** Expression of *ATP6AP2* in tumor tissues of either lung adenocarcinoma (LUAD) or lung squamous cell carcinoma (LUSC) are at similar levels to those of the corresponding normal tissues. TPM: transcripts per million; Correlation coefficient was analyzed via the Pearson-test

Recently, (P) RR has been demonstrated to play a pivotal role in the pathogenesis of PDAC. Shibayama et al. [11] demonstrated that compared with normal matched pancreatic tissues, premalignant pancreatic intraepithelial neoplasia (PanIN) and PDAC lesions exhibited aberrant (P) RR overexpression. Furthermore, (P) RR was revealed to be essential for Wnt/β-catenin-mediated proliferation of PDAC cells, while (P) RR silencing decreased Wnt signaling activity and cell proliferation, as well as induced the apoptosis of PDAC cells via caspase-3 activation [11]. Additionally, (P) RR expression was higher in human PDAC cell lines compared to that in normal pancreatic epithelial cells [11].To further analyze related information in big data, we have compared the levels of *ATP6AP2* transcripts in tumor tissues of pancreatic adenocarcinoma (PAAD) and normal pancreatic tissues, based on the data provided in the TCGA and GTEx databases. In consistence with previous reports, we found that *ATP6AP2* expression is significantly higher in tumor tissues than that in normal tissues (*P* < 0.01), moreover, level of *ATP6AP2* expression in PAAD is positively correlated with the expression level of *CTNNB1* (β-catenin encoding gene) and Wnt target gene *AXIN*2 (Fig. 4a). Soon after the finding of Shibayama et al., (P) RR was further definitively identified as a potential molecular biomarker for the diagnosis of PDAC by Arundhathi et al. [32], who showed that (P) RR mRNA level was positively correlated with TNM stage in human PDAC (patient number = 90, *r* = 0.688, *P* < 0.001) [32]. Moreover, anti-(P) RR antibody labelled with iodine-125 (^125^I) is a promising tracer for imaging diagnosis by single-photon emission computed tomography (SPECT)/CT in early stages of pancreatic cancer [32]. These findings revealed the essential roles of (P) RR in the molecular diagnosis and severity evaluation of pancreatic cancer.

#### Glioma

Kouchi et al. [13] reported that aberrant (P) RR overexpression was observed in WHO grade II–IV gliomas independent of IDH1 R132H mutation. Moreover, by detecting the tissue samples of 31 patients, they found that the (P) RR expression level was positively correlated with the WHO grade and Ki-67 labelling index (r = 0.8080, *P* < 0.0001), and negatively associated with the glioma patients’ survival times (r = − 0.6499, *P* < 0.0002) [13]. At the cellular level, (P) RR promoted the proliferation of human glioma cells through the Wnt/β-catenin pathway and inhibited cell apoptosis by attenuating caspase-3 activation [13]. Consistent with these findings, Juillerat-Jeanneret et al. [38] also reported a high expression level of (P) RR in human glioblastoma (GBM) (WHO grade IV), the most malignant type of glioma [39], and indicated that attenuation of renin activity in glioblastoma cells could decrease cell proliferation and induce apoptosis. To further analyze related information in big data, we have compared the levels of *ATP6AP2* transcripts in tumor tissues of GBM and normal matched tissues, based on the data provided in the TCGA and GTEx databases. In agreement with previous findings, we found that *ATP6AP2* expression is higher in tumor tissues than that in normal tissues, moreover, level of *ATP6AP2* expression in GBM is positively correlated with those of *CTNNB1, CCND1* and *AGT* (Fig. 4b). These findings suggest that (P) RR may contribute to glioma development through both Wnt signaling and the RAS signaling.

#### CRC

More than 80% of colorectal tumors harbor loss-of-function mutations in APC, and approximately 5% harbor activating mutation in β-catenin [40, 41], which result in constitutive aberrant activation of the Wnt/β-catenin pathway and thus promote carcinogenesis [42]. Interestingly, Voloshanenko et al. [43] found that Wnt3 knockdown noticeably reduced the activity of the Wnt/β-catenin pathway and proliferation of CRC cells independent of APC or β-catenin mutations. The above findings suggest that Wnts can further stimulate a mutated Wnt/β-catenin pathway with constitutively hyperactivated signaling activity. More recently, we have published the data that demonstrated, for the first time, that (P) RR promotes CRC through the Wnt/β-catenin pathway despite constitutive activating mutations in APC or β-catenin [10]. To further verify those results in big data, we have compared the levels of *ATP6AP2* transcripts in tumor tissues of colon adenocarcinoma (COAD) and normal matched tissues, based on the data in the TCGA and GTEx databases. In consistence with our previous findings, the results showed that *ATP6AP2* expression is higher in tumor tissues than that in normal tissues, moreover, level of *ATP6AP2* transcripts in COAD is positively correlated with those of *LRP6*, *CTNNB1, MYC, CCND1* and *AXIN2* (Fig. 4c). In addition, we further summarized the relationship between (P) RR expression levels, detected through the immunohistochemistry and evaluated by 3 independent pathologists as previously described [10], in primary tumor tissues and a series of detailed pathophysiological characteristics of 60 CRC patients (information collected from Kagawa University Hospital, Japan). We found that higher (P) RR expression levels are more commonly observed in patients with the following characteristics: male sex, early age at onset, poorly differentiated lesions, advanced cancer stage, distant metastasis, rapid progression, lower 5-year survival rate and shorter recurrence-free survival time (Table 1). Recently, Maider et al. [44] reported their latest data which are highly consistent with our previous findings. In their study, (P) RR protein levels were detected in tissues of adenomatous polyps and cancers from the same CRC patients (*n* = 42), as well as in tissues of primary tumors and nodal and liver metastases from advanced CRC patients (*n* = 294) [44]. The results showed that (P) RR expression increases throughout the colorectal adenoma—adenocarcinoma process, moreover, (P) RR protein in both primary tumor and distant metastases is associated with worse prognosis including 5- and 10-year survival of CRC patients [44].

**Table 1:** Association between (P) RR expression levels in primary colorectal tumors and clinicopathological characteristics

	Case number	(P) RR expression level (Immunohistochemistry)			Constituent ratio difference	
		**Weak**	**Middle**	**Strong**	***P*value**	**χ**^**2**^
		Case number (percentage)				
**Gender**	60					
**Male**	31	4 (12%)	15 (47%)	13 (41%)	< 0.0001***	27.36
**Female**	28	9 (32%)	16 (57%)	13 (11%)		
**Onset age**	60					
**<60**	12	1 (8%)	7 (59%)	4 (33%)	0.005**	10.6
**≥ 60**	48	12 (25%)	24 (50%)	12 (25%)		
WHO **Grade** of cancer differentiation	60					
**Grade 1**	17	5 (29%)	11 (65%)	1 (6%)	< 0.0001***	77.56
**Grade 2**	30	7 (23%)	16 (53%)	7 (23%)		
**Grade 3**	13	1 (8%)	4 (31%)	8 (61%)		
UICC **Stage**	60					
**Stage II**	20	9 (45%)	8 (40%)	3 (15%)	< 0.0001***	65.5
**Stage III**	20	3 (15%)	13 (65%)	4 (20%)		
**Stage IV**	20	1 (5%)	10 (50%)	9 (45%)		
**Distant Metastasis** and/or **Progression** after radical operation	60					
**No**	23	5 (22%)	14 (61%)	4 (17%)	0.0352*	6.695
**Yes**	37	8 (22%)	17 (46%)	12 (32%)		
**Five-year survival** (patients who finished the 5-year follow-up or died within 5 years)	47					
**Yes**	25	5 (20%)	13 (52%)	7 (28%)	0.0358 *	6.661
**No**	22	2 (9%)	11 (50%)	9 (41%)		
**Recurrence-free survival time** (duration from radical operation to recurrence) (months) (20 cases who had recurrence after radical operation)						
**(P) RR expression level**			***P*value**	**χ**^**2**^	**Hazard radio**	**95% CI**
**Week/Middle** (16 cases)	**Strong** (4 cases)					
10.4	5.85		0.0064**	7.438	0.06804	0.00986–0.4695

*UICC* The Union for International Cancer Control

Current therapeutic strategies for CRC are mainly limited to epidermal growth factor receptor (EGFR) monoclonal antibody-based treatment, which is focused on inhibiting the MAPK/ERK pathway (also called the EGFR-RAS-RAF-MEK-ERK pathway) and thus attenuates cancer cell proliferation. However, approximately 30–50% of CRCs carry a KRAS mutation, which automatically activates the downstream pathway and results in resistance to EGFR-targeted therapy. Interestingly, the close interaction between the Wnt/β-catenin and RAS-ERK pathways and the strong potential of anticancer strategies targeting Wnt signaling via the degradation of both β-catenin and RAS have been recognized [45]. Notably, Wang et al. [10] found that (P) RR silencing markedly inhibited the proliferation and induced the apoptosis of CRC cell lines carrying both activating mutations in Wnt signaling components and KRAS mutations DLD-1 (with APC and KRAS mutations) and HCT116 (with β-catenin and KRAS mutations) by weakening Wnt signaling activity. These findings suggest that (P) RR has great potential as a novel biomarker and therapeutic target for CRC despite spontaneously activating mutations in the Wnt/β-catenin and/or RAS-ERK pathways.

#### Breast cancer

Ohba et al. [14] previously indicated that (P) RR expression was detected by immunohistochemistry in breast cancer cells in 50 of 69 cases (72%) of breast carcinoma. Furthermore, the number of (P)RR-positive cases was much higher in the group with ≥10% Ki-67 (a cell proliferation marker) staining than in the group with < 10% Ki-67 staining. (P) RR silencing inhibited the proliferation of both MCF-7 (estrogen receptor (ER)α-positive) and SK-BR-3 (ERα-negative) breast cancer cells. By contrast, prorenin dose-dependently stimulated ERK1/2 phosphorylation in MCF-7 and SK-BR-3 cells [14]. Moreover, the proliferation of MCF-7 and SK-BR-3 cells was significantly decreased, to 32 and 44% that of the control cells, respectively, by treatment with 10 nM bafilomycin A1, an inhibitor of the V-ATPase [14]. The above data suggest that (P) RR may stimulate the proliferation of breast carcinoma cells, possibly via ERK1/2 phosphorylation and/or the association with the V-ATPase. We have also analyzed related information in the TCGA and GTEx databases. Consistently, we found that compared with normal matched tissues, the level of *ATP6AP2* transcripts in tumor tissues of breast invasive carcinoma (BRCA) are obviously higher, moreover, level of *ATP6AP2* expression in BRCA is positively correlated with those of the MAKP/ERK pathway components *KRAS* and *BRAF*, as well as that of the V-ATPase encoding gene *ATP6V1C2* (Fig. 4d). Therefore, (P) RR may contribute to the development of breast cancer through both the MAKP/ERK pathway and V-ATPase.

#### Aldosterone-producing adenoma (APA)

Recarti et al. [46] have detected high expression of (P) RR in APA tissues and HAC15 adrenocortical carcinoma cells that can produce aldosterone once treated with Ang II. Binding of prorenin and (P) RR could trigger the expression of the aldosterone synthase CYP11B2 and ERK1/2 phosphorylation, thus promoting the production and secretion of aldosterone [46]. Furthermore, Yamamoto et al. [15] revealed that both the mRNA and protein expression levels of (P) RR are elevated in tumor tissues of APAs relative to these levels in the matched non-neoplastic adrenal tissues and tissues from other adrenal tumors. These data suggest that (P) RR may play pivotal roles, such as promoting cell proliferation and aldosterone secretion, in APAs.

#### Endometrial cancer

Delforce et al. [28] revealed that a dysfunctional endometrial RAS aids the growth and spread of endometrial cancer. The protein and mRNA levels of RAS components in 30 human endometrial carcinomas and their corresponding adjacent endometrial tissues were measured, and data showed that protein levels of (P) RR and the mRNA levels of (P) RR, AGTR1, ACE1 and ACE2 were significantly higher in the tumor tissues than those in the matched normal endometrial tissues (*P* = 0.023, 0.008, 0.004 and 0.046, respectively) [28]. TGFβ1, a target of the endometrial RAS, was closely associated with RAS component expression and was upregulated in tumor tissues (*P* = 0.001) [28]. These data suggest that (P) RR possibly contributes to endometrial cancer by enhancing activity in the RAS. In addition, Lumbers et al. [47] further indicated that endometrial (P)RR/RAS system can be up-regulated by decidualization, increased prorenin will then stimulate the expression and secretion of vascular endothelial growth factor (VEGF), which could be important to establish an abundant blood supply for cancer development. However, there is a paradox when we analyzed related data in the TCGA and GTEx databases. Different from the findings of the above study, big data analysis showed that *ATP6AP2* transcripts in tumor tissues of uterine corpus endometrial carcinoma (UCEC) is at similar level to that of the matched normal tissues (Fig. 4e). Therefore, further study with larger sample number and further analysis of stratified samples are needed to explicit whether (P) RR indeed play a role in the pathogenetic process of endometrial cancer.

#### Lung cancer

Goldstein et al. [48] analyzed the data of lung cancer patients provided by the Gene Expression Omnibus (GEO) database (tumor: *n* = 57; normal tissue: *n* = 49) and found no significant difference in the *ATP6AP2* gene expression level between normal and lung cancer tissues. We have analyzed related information in the TCGA and GTEx databases and found that the *ATP6AP2* transcripts in tumor tissues of either lung adenocarcinoma (LUAD) or lung squamous cell carcinoma (LUSC) are slightly higher than those of the corresponding normal tissues (Fig. 4f). These findings suggest that (P) RR may not contribute much to lung cancer carcinogenesis.

### Future perspectives: applications in diagnosis and targeting

#### Plasma s(P)RR: aiding the prediction of cancer initiation and progression

Shibayama et al. [11] indicated that plasma s(P) RR levels in patients with PDAC are higher than those in healthy controls and that cultured human PDAC cell lines showed dramatically higher levels of s(P) RR secretion than normal pancreatic epithelial cells, indicating that plasma s(P) RR can be a potential and promising predictive biomarker for PDAC. However, the predictive value of plasmatic s(P) RR in CRC and primary epithelial ovarian cancer (EOC) seems not that obvious. Maider et al. [44] collected plasma from 161 patients with CRC followed by the measurement of s(P) RR, as a result, they found that plasmatic s(P) RR of CRC patients were at similar level to that of healthy controls and was not associated with disease progression. Additionally, Katrin et al. [49] tested s(P) RR in the plasma of 197 patients with primary EOC, analyzed the potential association between plasmatic s(P) RR and clinicopathological outcome of the patients, as well as compared levels of plasmatic s(P) RR of the patients and healthy controls. The results showed no correlation between s(P) RR level and clinicopathological characteristics of EOC including stage, grade and chemotherapy response, no association between s(P) RR level and prognostic parameters including overall survival and progression-free survival of patients, and no difference of s(P) RR level between patients and healthy controls [49]. In short, s(P) RR showed no predictive, prognostic, or diagnostic value in EOC. Hence, the indicative value of s(P) RR varies depend on different kinds of cancers. Therefore, future studies are needed to clarify whether plasma s(P) RR is also valuable in other kinds of cancers as a biomarker for early prediction, therapeutic evaluation and prognosis assessment. Notably, an s(P) RR ELISA kit has been developed to detect s(P) RR in blood and urine samples [50]. By using this assay, increased serum s(P) RR levels have been detected in patients with various diseases, including heart failure [51], kidney disease [52, 53], hypertension [54], and preeclampsia [55]. These results indicate the great feasibility of measuring plasma s(P) RR as a predictive biomarker in certain kinds of cancers.

#### Tumor tissue (P) RR expression: aiding cancer diagnosis, severity evaluation and prognosis prediction

Since the (P) RR expression levels in tumor tissues are closely correlated with the grades and stages of various cancers, (P) RR expression may serve as an adjuvant marker, in combination with current cancer-related protein markers, to aid diagnosing as well as evaluating the severity of and predicting the prognosis of various cancers.

#### Anti-(P) RR antibodies

**Fig. 5:**
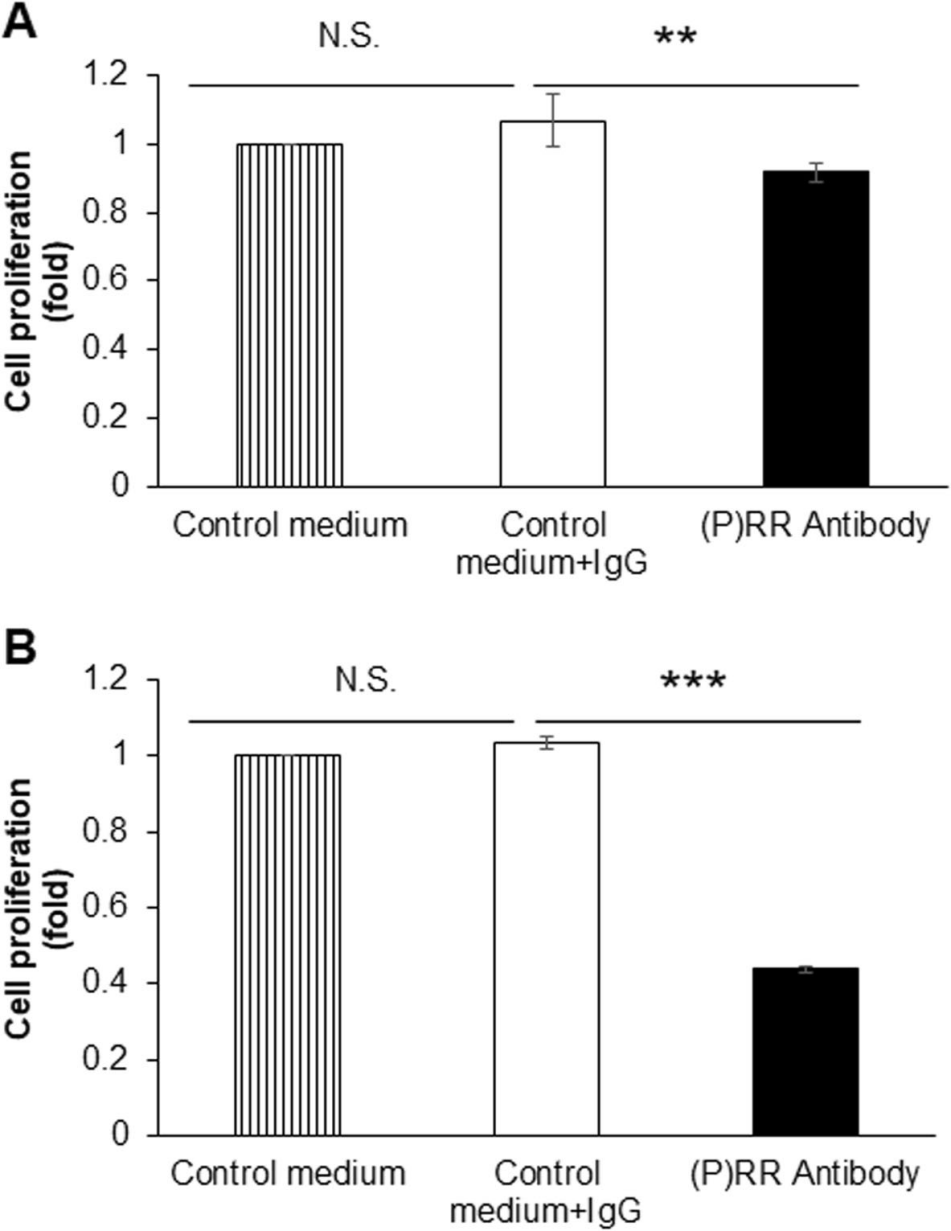
(P) RR inhibition attenuates the proliferation of CRC cells in vitro. **a** and **b** Treatment with (P) RR antibody for 48 h markedly inhibited the proliferation of DLD-1 (**a**) and HCT116 (**b**) cells, as measured by a WST-1 assay. The data are expressed as relative values compared to the vehicle-treated group. N.S.: not significant. **P < 0.01. ***P < 0.001. *n* = 4

Since (P) RR intensely promotes many kinds of cancers, overcoming cancers with anti-(P) RR monoclonal antibodies should be very promising. We have generated a monoclonal antibody against (P) RR targeted to the region spanning amino acids 200 to 213 in the extracellular domain of (P) RR and then confirmed its specificity and quality, in a way described previously [56]. To assess the effect of this antibody, human CRC DLD-1 and HCT116 cells, purchased from the American Type Culture Collection (ATCC; Manassas, VA, USA), were seeded into 24-well plates at a density of 2 × 10 [4] cells/well. After the cells grew to approximately 40% confluence, they were serum-starved for 24 h without antibiotics and were then treated with either the monoclonal (P) RR antibody or human IgG (Thermo Fisher Scientific, catalogue #31154) as the negative control at a concentration of 200 μg/mL (500 μL of solution per well). After treatment for 48 h, cell proliferation activity was assessed by a water-soluble tetrazolium salt (WST)-1 assay, as previously described [10]. The WST-1 assay data indicated that cell proliferation was consistently attenuated by blocking the specific amino acid regions on the extracellular domain of (P) RR with the monoclonal antibody. Compared with treatment with human IgG, treatment with the (P) RR antibody for 48 h markedly decreased the proliferation of both DLD-1 (mean ± SEM; 1.07 ± 0.08 vs. 0.92 ± 0.03, relative ratio compared to the vehicle group without antibody treatment; *P <* 0.01) (Fig. 5a) and HCT116 (mean ± SEM; 1.04 ± 0.02 vs. 0.44 ± 0.01, relative ratio compared to the vehicle group; *P <* 0.001) cells (Fig. 5b). These data revealed the promising potential anti-colon cancer effect of the (P) RR antibody, and further studies on (P) RR antibodies are ongoing.

## Conclusions

Overexpression of (P) RR, which may contribute to cancer initiation and progression via the Wnt/β-catenin, RAS, MAPK/ERK and PI3K/AKT/mTOR pathways as well as V-ATPase function, has been observed in various cancers including pancreatic, brain, colorectal, endometrial, breast and adrenal cancers (Table 2). Since multiple functions of (P) RR play important roles in cancer pathophysiology, alternative pathways will also be identified in the future. Taken together, these findings indicate that (P) RR, including the full-length form in tumor tissues and the soluble form in the blood, has substantial potential as a novel biomarker for cancer diagnosis, severity evaluation and prognosis prediction and is also a promising therapeutic target for cancers. Therefore, (P) RR monoclonal antibodies, which are in development and testing, will play essential roles in cancer treatment in the future.

**Table 2:** Studies investigating the roles of (P) RR in different cancers

Study	Tumor type	Cancer cell line (human)	(P)RR-Related pathway or factor	Finding
Shibayama et al., 2015 [11]	Pancreatic ductal adenocarcinoma (PDAC)	PK-8, PCI-35, BxPC-3, PK-1, PANC-1 and MIAPaCa-2	Wnt/β-catenin pathway	Higher (P) RR expression: higher proliferation ability and less apoptosis of cancer cells; enhanced Wnt/β-catenin activity
Arundhathi et al., 2016 [32]	PDAC	Panc-1, ASPC, BXPC-3, HPAC, and MIAPaCa-2	MAPK/ERK and PI3K/AKT/mTOR pathways	Higher (P) RR expression: more advanced disease; enhanced MAPK/ERK and PI3K/AKT/mTOR signaling activity
Kouchi et al., 2017 [13]	Glioma	U251MG, U87MG, and T98G	Wnt/β-catenin pathway	Higher (P) RR expression: more advanced disease; higher proliferation ability and less apoptosis of cancer cells; enhanced Wnt/β-catenin activity
Juillerat-Jeanneret et al., 2004 [38]	Glioblastoma (GBM)	LN18 and LNZ308	RAS pathway	Higher (P) RR expression: more advanced disease; higher proliferation ability of cancer cells; enhanced RAS activity
Wang et al., 2019 [10]	Colorectal cancer (CRC)	DLD-1 and HCT116	Wnt/β-catenin pathway	Higher (P) RR expression: higher proliferation ability and less apoptosis of cancer cells; enhanced Wnt/β-catenin activity
Maider et al., 2019 [44]	CRC	Not applicable (only clinical samples were used)	Not applicable	Higher (P) RR expression: more advanced disease; worse prognosis (e.g. 5- or 10- year survival)
Ohba et al., 2014 [14]	Breast cancer	MCF-7, T47D, SK-BR-3 and MDA-MB-231	MAKP/ERK pathway and V-ATPase	Higher (P) RR expression: higher proliferation ability of cancer cells
Yamamoto et al., 2013 [15]	Aldosterone-producing adenoma (APA)	H295R and HAC15	Not mentioned	Higher (P) RR expression: higher proliferation ability of cancer cells and more aldosterone secretion
Delforce et al., 2017 [28]	Endometrial cancer	MCF-7	RAS	Higher (P) RR expression: higher proliferation ability of cancer cells, possibility of cancer spread and RAS activity

## Data Availability

The material supporting the conclusion of this review has been included within the article.

## Abbreviations

(P)RR: (pro) renin receptor
ACE: angiotensin-converting enzyme
AGT: angiotensinogen
AGTR: Ang II/Ang II receptor
Ang: angiotensin
APC: Adenomatous polyposis coli
Axin: axis inhibition protein
CCND1: cyclin D1
COAD: colon adenocarcinoma
CRC: colorectal cancer
CTNNB1: catenin beta 1
DLBC: lymphoid neoplasm diffuse large B-cell Lymphoma
EMT: epithelial-to-mesenchymal transition
EOC: epithelial ovarian cancer
ERK: extracellular signal-regulated kinases
ERK1/2: extracellular signal-regulated kinase 1/2
FL(P)RR: full-length (P)RR
GBM: glioblastoma
GEO: Gene Expression Omnibus
GSK3: Glycogen synthase kinase 3
GTEx: Genotype-Tissue Expression
KIRC: kidney renal clear cell carcinoma
LEF: lymphoid enhancer-binding factor
LRP6: low-density lipoprotein receptor protein 6
LUAD: lung adenocarcinoma
LUSC: lung squamous cell carcinoma
MAPK: mitogen-activated protein kinase
mTOR: mammalian target of rapamycin
PAAD: pancreatic adenocarcinoma
PAAD: pancreatic adenocarcinoma
PanIN: pancreatic intraepithelial neoplasia
PDAC: pancreatic ductal adenocarcinoma
RAS: renin-angiotensin system
ROS: reactive oxygen species
s(P)RR: soluble (P)RR
SPECT: single-photon emission computed tomography
STAD: stomach adenocarcinoma
TCF: T-cell factor
TCGA: Cancer Genome Atlas
TGCT: testicular germ cell tumors
TGF: transforming growth factor
THYM: thymoma
UCEC: uterine corpus endometrial carcinoma
V-ATPase: vacuolar H + -ATPase
VEGF: vascular endothelial growth factor
WST: water-soluble tetrazolium salt
